# Alteration of Intestinal Flora Stimulates Pulmonary microRNAs to Interfere with Host Antiviral Immunity in Influenza

**DOI:** 10.3390/molecules23123151

**Published:** 2018-11-30

**Authors:** Peng Pang, Bin Yu, Yucong Shi, Li Deng, Huachong Xu, Sizhi Wu, Xiaoyin Chen

**Affiliations:** 1 College of Traditional Chinese Medicine, Jinan University, Guangzhou 510632, Guangdong, China; pangpeng@stu2014.jnu.edu.cn (P.P.); yubinsd@yeah.net (B.Y.); shiyucong@stu2016.jnu.edu.cn (Y.S.); dengli@stu2015.jnu.edu.cn (L.D.); xuhuachong@stu2015.jnu.edu.cn (H.X.); wusizhi@stu2015.jnu.edu.cn (S.W.); 2 College of Integrated Traditional Chinese and Western Medicine, Jining Medical University, Jining 272067, Shandong, China

**Keywords:** intestinal flora, microRNAs, influenza, antibiotic abuse, antiviral immunity

## Abstract

The intestinal flora may be an important and modifiable factor that contributes to the immune response in influenza. To investigate the effect of intestinal flora alteration induced by antibiotic interference on microRNA (miRNA) communication in antiviral immunity, BALB/c mice received two weeks of antibiotic treatment before infection with the influenza A virus. The changes in intestinal flora and pulmonary flora were detected and analyzed by 16S ribosomal RNA (rRNA) gene sequencing. The amplification of the influenza virus in the lungs was measured by RT-PCR. The involvement of pulmonary miRNA was explored using miRNA microarray analysis. The results showed that the antibiotics destroyed the symbiotic relationship of the intestinal flora, resulting in a reduction in bacterial diversity, but they did not affect the pulmonary flora. The alteration of intestinal flora affected the expression of pulmonary miRNAs and resulted in an enhancement of pulmonary influenza virus amplification. The conclusion is that alteration of intestinal flora induced by antibiotic interference affected the expression of pulmonary miRNAs to interfere with host antiviral immunity, of which miR-146b and miR-29c might be good resources of resistance to influenza under antibiotic abuse.

## 1. Introduction

Influenza is a heavy burden of global public health because of its strong infectivity and high morbidity. The most common symptoms of influenza include cough, fever, headache, and weakness [1]. In addition, gastroenteritis-like symptoms, such as nausea, vomiting, abdominal pain, and diarrhea, often accompany influenza in patients [2]. Influenza virus infection is believed to cause changes in intestinal flora, which might be responsible for the changes in excrement characteristics [3]. What is worse, the abuse of antibiotics often exacerbates changes in the gut microbiome. Therefore, the alteration of intestinal flora induced by antibiotic interference may be an important and modifiable factor that contributes to the immune response in influenza.

Accumulating evidence suggests that commensal bacteria have a crucial impact on the host’s immune system [4]. On one hand, intestinal flora and the host immune system jointly defend the invasion of pathogenic microorganisms. The healthy intestinal microenvironment contributes to the maintenance of the immunological status of the host. On the other hand, the deviations in microbial community composition increase the risk of pathogen infection, which can cause disease or influence its course and prognosis [5]. Dysbacteriosis of the gut microbiota could cause damage to the gastrointestinal mucosal barrier, dysfunction of antibody synthesis or secretion, and development of diseases in the respiratory system. For instance, the intestinal microbiota is related to chronic intestinal inflammation in various inflammatory bowel diseases (IBDs) [6]. What is more, studies in recent years also testified that the intestine microbiota affected the host’s immune response to extragastrointestinal tract infections [7]. The intestinal microbiota of the host affects the replication and transmission of a diverse array of viral pathogens, such as influenza virus, dengue virus, and human immunodeficiency virus [8]. For example, Ichinohe et al. revealed that alteration of intestinal flora resulted in defective cluster of differentiation 4 (CD4) T-cell, CD8 T-cell, and B-cell immunity in influenza virus infection [9]. Abt et al. demonstrated that intestinal dysbacteriosis altered the expression of antiviral defense genes and interfered in the responsive pathways, which led to the failure to control viral infection and an increase in host morbidity and mortality [10]. In addition, our previous study also found that dysbacteriosis downregulated the Toll-like receptor 7 (TLR7) signaling pathway, which affected the immunity of the respiratory mucosa against influenza A virus infection [11]. Therefore, the alteration of the intestinal flora might interfere with the host immune function. 

However, due to the complexity and diversity of commensal–host interactions, more study is needed to increase the knowledge of the joint modulating mechanism of immune homeostasis and intestinal microbiota. Interdomain molecular communication plays an important role in host and gut microorganism interactions, of which microRNAs (miRNAs) are considered to be a key mode [12]. MicroRNAs have many regulatory roles in various cellular processes and they are implicated in various diseases. Researchers claimed that the microbiota regulated dendritic cell (DC) expression of interleukins 12 and 23p40 (IL-12/IL-23p40) through the inhibition of miR-10a, which could be important in the maintenance of host immune homeostasis [13]. Therefore, the relationship between intestinal flora and miRNA expression should be discussed. The mechanism of the intestinal microbiota that affects the natural immunoreaction in influenza infection is interesting; however, the roles of the intestinal flora and microRNA communication in the influenza immune response are yet to be clarified. Therefore, in this study, mice were treated with antibiotics before viral infection to establish an animal model of dysbacteriosis, to investigate the mechanism of intestinal flora and miRNAs in the immune response to influenza.

## 2. Materials and Methods

### 2.1. Animals

Forty-eight SPF (Specefic pathogen Free, SPF) BALB/c mice, half males and half females, weighing 16–18 g, were purchased from Guangdong Medical Laboratory Animal Center (Guangdong, China). The mice were kept in the SPF laboratory animal room in a controlled environment (22 ± 1 °C and 55% relative humidity) with free access to food and water during the experiment. The experiment was performed under the supervision and assessment of the Laboratory Animal Ethics Committee of Jinan University. Every experiment operation abided by the statute on the Administration of Laboratory Animals approved by the China Council in 1988.

### 2.2. Grouping and Treatment

**Figure 1:**
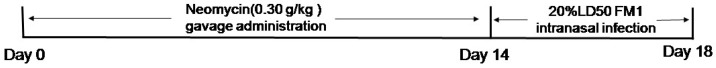
Time order of treatment for mice.

After sample size calculating, the mice were randomly divided into four groups: control group (C), virus infection model group (V), dysbacteriosis model group (D), and dysbacteriosis and virus infection group (D + V). Neomycin (0.30 g/kg, dissolved in normal saline (NS) administered via gavage for 14 days) was used to establish an intestinal dysbacteriosis mouse model in the D and D + V groups. The C and V groups received NS intragastrically as a comparison. The influenza A virus (A/FM1/1/47) was provided by the Department of Immunology and Microbiology of Jinan University (Guangzhou, China). After full anesthetization by inhalation of diethyl ether, 20% median lethal dose (LD_50_) FM1 virus intranasal infection for four days was used to establish the virus infection model in the V group and D + V group from the 15th day of the experiment. Intranasal treatment with NS was also performed in the C and D groups to avoid the impact of the operation. All the mice were sacrificed on the 19th day after abstaining from food except water for 12 h (Figure 1).

### 2.3. 16S rRNA for Intestinal Flora

After the mice were sacrificed, all the stool samples were collected from the cecum into sterile centrifuge tubes. Bacterial genomic DNA was extracted using the QIAamp Fast DNA Stool Mini Kit (Cat: 51604, Qiagen, Germany) according to the manufacturer’s protocol. Then, 16S rRNA gene sequencing was carried out by BGI-Shenzhen, Shenzhen, China. A quality test was performed before the qualified DNA was used to construct a library. The 16S rDNA V4 region was amplified by polymerase chain reaction (PCR) and the primers flanking the hypervariable V4 region were 5′-AAATTTTTTTTCCCCCCCGG-3′ (forward primer) and 5′-GGGCCCCCTTTAAAAAAAAAC-3′ (reverse primer). The 2 × 250-bp paired-end reads were generated with the Illumina MiSeq platform (San Diego, CA, USA). To obtain clean reads, the raw data were filtered to eliminate the adapter pollution N base, poly-bases, and low-quality sequencing reads. The high-quality paired-end reads were combined with tags based on overlaps, and then the tags were clustered into operational taxonomic units (OTUs) at 97% sequence similarity by USEARCH (v7.0.1090, http://www.drive5.com/usearch/). OTU representative sequences were taxonomically classified using the Ribosomal Database Project (RDP) Classifier v.2.2 trained on the Greengenes database (v201305, http://greengenes.secondgenome.com/downloads). Finally, the alpha diversity and the different species screening were analyzed based on OTU and taxonomic ranks. Software R (v3.1.1, http://www.r-project.org/) was used to draw the Venn diagram, PCA (principal component analysis, PCA, package “ade4”), and the histogram of family taxonomic rank. The species heatmaps were generated using the package “gplots” of software R (v3.1.1). The distance algorithm was “Euclidean”, and the clustering method was “complete”.

### 2.4. 16S rRNA for Lung Flora

A portion of lung tissue was dissected after the mice were euthanized. Pulmonary bacterial genomic DNA extraction and 16S rRNA gene sequencing were carried out by BGI-Shenzhen, Shenzhen, China. The specific detection methods are as described in Section 2.2. A heat map and a histogram of genus taxonomic rank were generated using software R (v3.1.1).

### 2.5. Lung Index and Virus Quantification

The animals were weighed before being sacrificed. The lung tissue of mice was removed and washed with normal saline (NS). After soaking up the water on the surface with a filter, the lung tissue was weighed with an electronic balance. The following formula was used to calculate the lung index: lung index = (lung weight/body weight) × 100%.

Approximately 20 mg of lung tissue was separated from the lung, and the total RNA was extracted with 1 mL of Beyozol reagent (Beyotime Biotechnology, Haimen, China). Nucleic acid concentrations were quantified with a Thermo Nanodrop ND-1000 spectrophotometer (Rockland, DE, USA). The purity of RNA samples was assessed by measuring the optical density ratio at 260 and 280 nm (OD260/OD280). The RNA of each sample was diluted to the same concentration and was reverse-transcribed to complementary DNA (cDNA) using the Reverse Transcription Kit (Tiangen, Biotech Co. Ltd, Beijing, China) according to the manufacturer’s protocol. The viral RNA expression was examined using the real master mix (Tiangen Biotech Co. Ltd, Beijing, China). PCR was performed, and the amplification products were analyzed on a Bio-Rad Mini-Opticon detection system (#CPB3120EDU, Hercules, CA, USA). Glyceraldehyde-3-phosphate dehydrogenase (GAPDH) was taken as an internal parameter and the viral primer sequences were designed according to the hemagglutinin (HA) gene of influenza A virus. The forward primer sequence of the virus was 5′-GACCAATCCTGTCACCTCTGAC-3′, and the reverse primer sequence was 5′-GGGCATTTGGACAAACGTCTACG-3′. The primers were synthesized by Generay Biotech Co., Ltd, Shanghai, China. The protocol was as follows: 1 min at 95 °C, followed by 40 cycles of 15 s at 94 °C, 30 s at 60 °C, and 30 s at 72 °C. After RT-qPCR, the 2^−ΔΔCT^ method was used for analysis of the relative virus amplification. Each sample was run in triplicate and averaged to ensure accuracy. 

### 2.6. Microarray Analysis, RT-PCR Validation, and Prediction of Differentially Expressed microRNA Targets

RNA samples were isolated from the lungs of groups C, V, D, and D + V. Microarray analysis of RNA was performed using an Affymetrix Mouse Command Console Chip v4.0 (OE Biotech. Co., Ltd., Shanghai, China). Three biological replicates were processed from each group. The raw data from the miRNA-chip were normalized separately using Agilent GeneSpring GX v12.5 (Santa Clara, CA, USA). Both the on chip and on-gene median methods were used to normalize the gene expression data. Microarray data were then joined into one GeneSpring genome, and samples were assigned to one of two groups. Probes that had at least 100.0% of samples in any one out of two conditions that had flags in “*p*-value” were chosen for further analysis. Differentially expressed miRNA was then identified through fold change, as well as the *p*-value calculated using the *t*-test. The threshold set for up- and downregulated genes was a fold change ≥ 1.5 and a *p*-value ≤ 0.05. Hierarchical clustering was performed to show the distinguishable miRNA expression pattern among samples.

The miRNAs that changed in all four groups were screened, and the expression levels of all samples were verified by RT-qPCR. The quantification was performed with reverse transcription (RT) and PCR. Each RT reaction consisted of 1 μg of RNA, 4 μL of miScript HiSpec Buffer, 2 μL of Nucleics Mix, and 2 μL of miScript Reverse Transcriptase Mix (Qiagen, Duesseldorf, Germany), for a total volume of 20 μL. Reactions were performed in a GeneAmp^®^ 9700 PCR System (Applied Biosystems, Waltham, MA, USA) for 60 min at 37 °C, followed by heat inactivation of RT for 5 min at 95 °C. The conditions for RT-qPCR were incubation at 95 °C for 10 min, followed by 40 cycles of 95 °C for 10 s, and 60 °C for 30 s. The miRNA-specific primer sequences were designed based on the miRNA sequences obtained from the miRBase database (Release 20.0, http://www.mirbase.org/) and synthesized by Generay Biotech (Shanghai, China). Expression analysis was performed in triplicate for each sample. The expression levels of miRNAs were normalized to 18S rRNA and were calculated using the 2^−ΔΔCt^ method. Furthermore, target genes of differentially expressed miRNAs were predicted using the intersection of the TargetScan (http://www.targetscan.org/vert_71/) and microRNA.org databases (http://www.microrna.org/microrna/microrna/home.do). Target gene prediction was performed using the microRNA.org (version 2012.06.10, default parameters, conservation > 0.25, mirSVR score > −0.1) and microRNA.org (version 2016.06, context score > 50%) databases. 

### 2.7. Statistical Analysis

Data analysis was performed with SPSS for Windows (version 19, IBM Software, New York, NY, USA). The significance of differences between groups was evaluated using one-way ANOVA, and the differences were considered statistically significant at *p* < 0.05. All values are represented as the means ± SD. 

## 3. Results

### 3.1. OTU Abundance Differences

**Figure 2:**
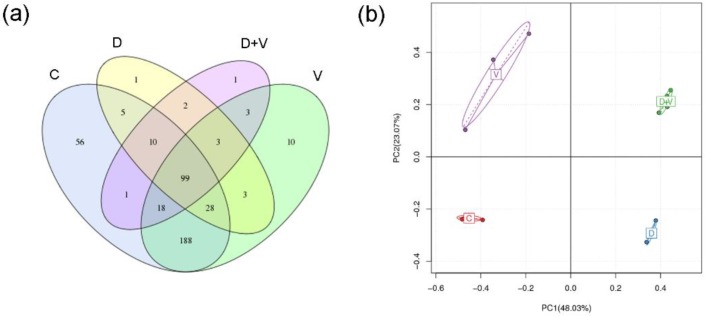
Operational taxonomic unit (OTU) cluster and abundance. (**a**) OTU Venn chart. Different colors represent different groups. The interior of each circle symbolically shows the number of observed OTUs in the specific group, and the intersection represents the set of OTUs commonly present in the counterpart groups. (**b**) Principal component analysis (PCA) based on OTU abundance (description). The *x*-axis represents the first principal component and the *y*-axis represents the second principal component. The number in brackets represents the contributions of principal components to differences among samples. Each sample was shown by a dot with its group color. The distance between the points in the two-dimensional (2D) graph shows their similarity, that is, the closer points had similar microbiological compositions. Group designations were as follows: control (C); virus infection model (V); dysbacteriosis model (D); dysbacteriosis and virus infection (D + V).

The OTU is an operational definition of a species or species group that is used to define the species distinction in microbiology. The OTU Venn chart was used to represent the intersection relationship between groups, which can visually display the number of communal and exclusive OTUs in different groups. The common OTU number of the four groups was only 99, which suggested that there were obvious differences in microbiological composition among the four groups. In addition, the common OTU number between group C and group V was 333, while it was 142 between group C and D, and 128 between group C and D + V. This suggested that the microbiological composition in group D + V changed most compared with group C, followed by group D and group V (Figure 2a).

PCA was used to summarize factors mainly responsible for the differences in OTU composition in different samples. When several samples from the same group show a kind of aggregated distribution pattern, it implies a high homogeneity in the individual group. The distance between different groups displays the variance of OTU composition between groups. According to the aggregation distribution of samples within groups, the results of PCA showed that the samples in groups C, D, and D + V had better homogeneity. The four groups were separated into four quadrants, which represented the distinct variance of OTU composition among the four groups. Therefore, the results of the OTU Venn chart and PCA showed that there were significant differences in OTU numbers and composition in the four groups (Figure 2b).

**Figure 3:**
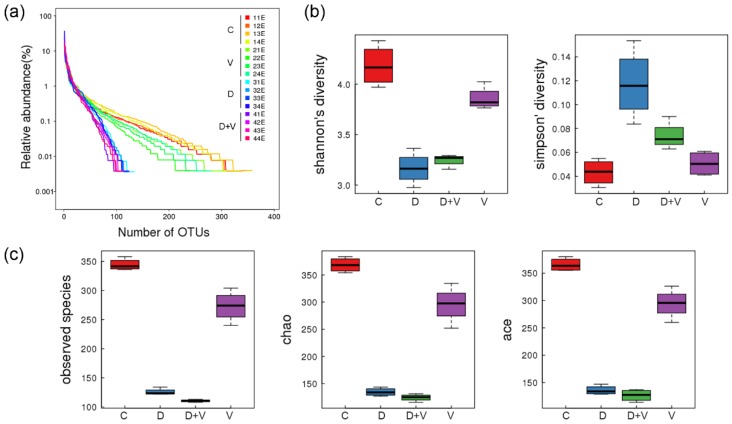
Diversity analysis. (**a**) OTU rank curve of each sample. The width of the *x*-axis in the chart displays the species richness, while the species evenness is derived from the slope of the line. A longer curve on the horizontal axis means the species is more abundant in the sample. A flatter curve indicates that the species composition of the sample is more homogeneous. The smoothness of the curve shows the diversity of the sample. A smooth curve indicates a sample with high diversity, while a rapidly descending curve implies a high proportion of dominant bacteria and low diversity. (**b**) Boxplots of the Shannon and Simpson indexes of alpha diversity indexes among groups to reflect the species diversity of the community. (**c**) Boxplots of the observed species, Chao, and Ace indexes of alpha diversity indexes among groups to reflect the species richness of community. The complexity of the sample is proportional to the observed species, Chao, Ace, and Shannon indexes, with a negative correlation with the Simpson value. When the species richness is the same, the greater the species evenness, and the greater the community diversity will be. Group designations were as follows: control (C); virus infection model (V); dysbacteriosis model (D); dysbacteriosis and virus infection (D + V).

The OTU rank abundance curve provides an intuitive performance of both species richness and species evenness. The normal control group C had the highest species richness and species evenness, because it had the maximum width of the *x*-axis and the smallest slope. Group V also had a relatively smooth curve, which indicated a relatively high diversity of samples. Groups D and D + V had a greater slope, which implied low diversity, which might be due to a high proportion of dominant bacteria. Thus, dysbacteriosis led to an evident negative effect on species richness and species evenness, and it could be a little worse when combined with influenza virus infection. (Figure 3a).

Alpha diversity is used for analyzing the species diversity in individual groups, and the evaluation indexes include Shannon, Simpson, observed species, Chao, and Ace. On one hand, the indexes, observed species, Chao, and Ace, reflect the species richness of community. The index observed species shows the number of OTUs classified. Chao stops sampling until no more Singleton (the number in samples = 1) is found. Ace takes into account Singleton and the species that appear ≤10 times to estimate the species that have not been discovered. Thus, the higher the observed species, Chao, and Ace indexes represent the higher species richness of community (Figure 3c). On the other hand, Shannon and Simpson indexes show both the richness and evenness of species to reflect the species diversity of the community. Simpson looks at the probability that two randomly sampled individuals belong to the same species. Shannon analyzes the indeterminacy of the collected species. Therefore, the complexity of the sample is proportional to Shannon and has a negative correlation with the Simpson value (Figure 3b).

The results of alpha diversity showed that group C had the highest values in observed species, Chao, Ace, and Shannon and the lowest value in Simpson, which indicated that the normal group has the highest diversity of species. In the indexes of observed species, Chao, and Ace, the values from high to low were as follows: group C > group V > group D + V > group D, which suggested the species richness order of the four groups. However, the results showed by Shannon and Simpson were that group D had the lowest diversity of species, followed by group D + V. When the species richness is the same, the greater the species evenness is, the greater the community diversity will be. Thus, the difference indicated that group D + V had a greater species evenness than group D. Therefore, dysbacteriosis significantly affects species richness of intestinal bacteria, with the largest impact on the D + V group. Furthermore, the species diversity from high to low was as follows: group C > group V > group D + V > group D (Figure 3b,c).

### 3.2. Changes in Composition and Structure of Intestinal Flora

**Figure 4:**
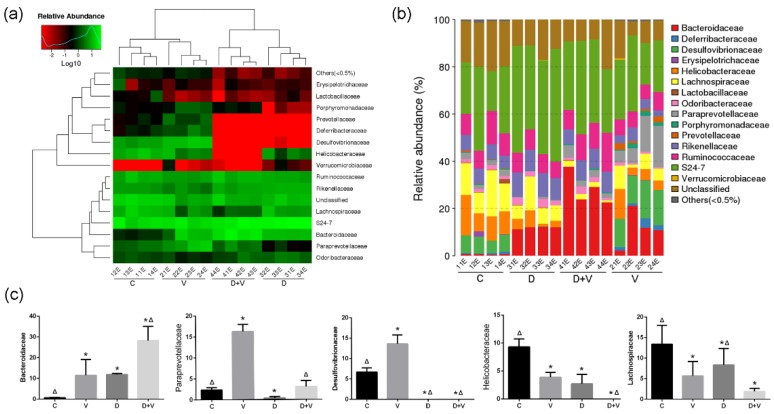
Species composition and abundance of intestinal flora. (**a**) Log-scaled percentage heat map at the family level. Longitudinal clustering indicates the similarity of all species among different samples, while the horizontal clustering indicates the similarity of certain species among different samples. The closer distance and the shorter branch length represent the similarity of the species composition between the samples. (**b**) The taxonomic composition distribution in samples at the family level. The taxonomic composition distribution histograms of each sample were shown at the family level and directly displayed as the ratio of each species. The species with abundance lower than 0.5% were classified into “others”. (**c**) Statistical results of microflora with significant changes at the family level, including Bacteroidaceae, Paraprevotellaceae, Desulfovibrionaceae, Helicobacteraceae, and Lachnospiraceae. * *p* < 0.05 compared with group C; ^Δ^ *p* < 0.05 compared with group V. Group designations were as follows: control (C); virus infection model (V); dysbacteriosis model (D); dysbacteriosis and virus infection (D + V).

A log-scaled percentage heat map and taxonomic composition distribution in samples at the family level were used to show the species composition of the whole community at the family level in each sample and the differences among them (Figure 4a,b). In order to show the differences of species composition and structure more intuitively, five bacteria with significant changes were chosen and displayed in the histogram (Figure 4c). Using RNA sequencing (RNA-seq) analysis of 16S rRNA, it was found that neomycin treatment decreased the relative abundance of Paraprevotellaceae, Desulfovibrionaceae, Helicobacteraceae, and Lachnospiraceae, and increased the Bacteroidaceae in the murine colon microbiota, compared with the normal group. The imbalance of the ratio of Gram-positive to Gram-negative bacteria showed that the model of intestinal flora alteration was successfully established. Influenza virus resulted in significantly increased cecal bacterial counts in Bacteroidaceae, Paraprevotellaceae, and Desulfovibrionaceae, while counts in Helicobacteraceae and Lachnospiraceae significantly decreased. However, when the virus infection combined with intestinal dysbacteriosis, the bacterial counts showed some interesting variations. In the D + V group, Bacteroidaceae significantly increased compared to group V, while Helicobacteraceae and Lachnospiraceae showed a negative trend. In addition, contrary to the increase in group V, Paraprevotellaceae and Desulfovibrionaceae were reduced and even disappeared in group D + V. 

### 3.3. Minimal Changes in Composition and Structure of Pulmonary Flora

**Figure 5:**
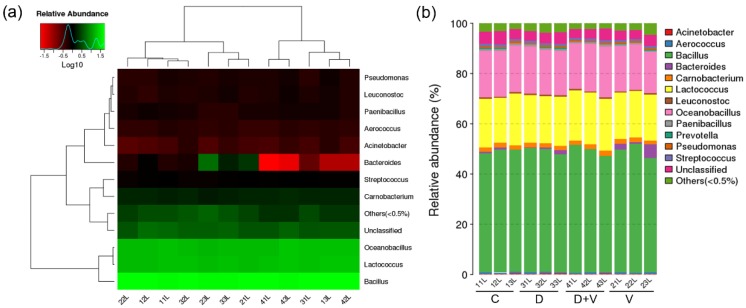
Microbial diversity and species abundance of pulmonary flora. (**a**) Log-scaled percentage heat map at the genus level. Longitudinal clustering indicates the similarity of all species among different samples, while the horizontal clustering indicates the similarity of certain species among different samples. The closer distance and the shorter branch length represent the similarity in the species composition between the samples. (**b**) The taxonomic composition distribution in samples at the genus level. The taxonomic composition distribution histograms of each sample were shown at the genus level and directly displayed as the ratio of each species. The species where abundance was lower than 0.5% were classified into “others”. Group designations were as follows: control (C); virus infection model (V); dysbacteriosis model (D); dysbacteriosis and virus infection (D + V).

To observe the effect of antibiotics and virus intervention on pulmonary flora, RNA-seq analysis of 16S rRNA of lung flora was carried out. For different groups, the heat map on species abundance could visually reflect the small difference among groups (Figure 5a). The heat map showed species clustering based on the abundance of each species. The results of the heat map showed that the samples belonging to the same group were not clustered together, which indicated that the samples of the four groups had similar species composition. In addition, although *Bacteroides* showed a color difference, it had no statistical significance. Thus, based on the longitudinal clustering and similar colors, the heat map manifested no intergroup difference. The taxonomic composition distribution in samples at the genus level showed the type and proportion differences in species (Figure 5b). The four groups had similar species structure and composition. The major components of the pulmonary flora were *Bacillus*, *Lactococcus*, and *Oceanobacillus*. The results indicated that the composition and structure of pulmonary flora among the four groups had no significant differences. Therefore, both antibiotic intervention and influenza virus infection had little effect on pulmonary flora.

### 3.4. Changes in Lung Index and Virus Amplification in the Lung

**Figure 6:**
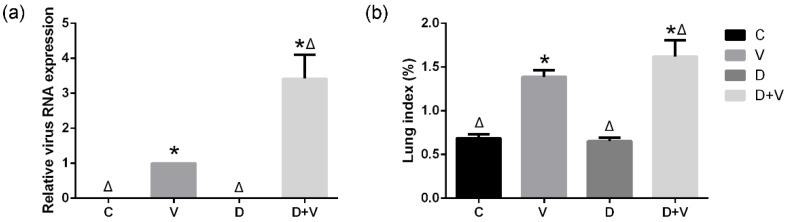
Relative expression of viral RNA in the lung and the lung index. (**a**) Relative expression of viral RNA in the lung. Each sample was run in triplicate and averaged to ensure accuracy. The 2^−ΔΔCT^ method was used for analysis of the relative virus amplification. The values of groups C, D, and D + V represent the fold change relative to group V which was converted to 1. (**b**) Lung index. The formula used was as follows: lung index = (lung weight/body weight) × 100%. * *p* < 0.05 compared with group C; ^Δ^ *p* < 0.05 compared with group V. Group designations were as follows: control (C); virus infection model (V); dysbacteriosis model (D); dysbacteriosis and virus infection (D+V).

Virus amplification in the lungs was used to define whether the virus model was successfully established and to differentiate the degree of severity of the virus infection. According to the PCR results, group C and group D showed no virus expression, while virus amplification in groups V and D + V was significantly increased. This indicated that the mouse virus model was successfully established. Furthermore, the expression of group D + V (3.42 ± 0.72) was significantly higher than that of group V, which suggested that intestinal dysbacteriosis might influence the antiviral function of the host (Figure 6a).

In general, severe pulmonary infection can cause pulmonary edema, which increases the lung weight. The lung indexes of group C and group D were similarly and had no statistical significance. However, the influenza virus infection increased the weights of the lungs and decreased the body weights of mice, which resulted in a high lung index. Thus, the lung index in group V increased significantly compared to group C. What is more, group D + V (1.62 ± 0.19) had a higher lung index compared to group V (1.39 ± 0.08), which implied a more serious pulmonary infection in group D + V (Figure 6b). 

### 3.5. Screening of Differentially Expressed miRNA

**Figure 7:**
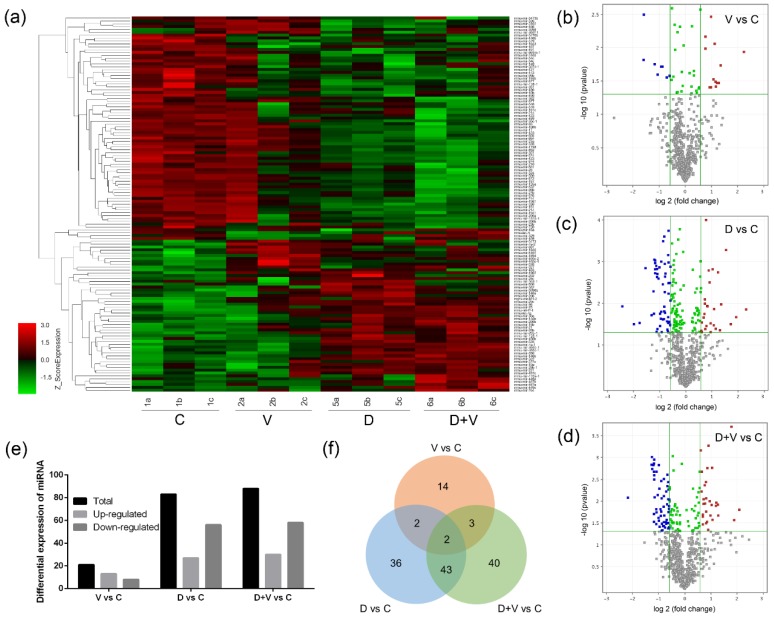
Changes in microRNA (miRNA) expression profiles as indicated by miRNA microarray analysis. (**a**) Heat map of the union of differentially expressed miRNAs between group V vs. C, group D vs. C, and group D + V vs. C. (**b**) Volcano plots of differentially expressed miRNAs between groups V and C. (**c**) Volcano plots of differentially expressed miRNAs between groups D and C. (**d**) Volcano plots of differentially expressed miRNAs between groups D + V and C. (**e**) Histogram of differential expression of miRNAs. (**f**) Venn chart of the intersection of differentially expressed miRNAs between group V vs. C, group D vs. C, and group D + V vs. C. In the Figure 7b–d, the blue dots represent the downregulated miRNA expression (fold change ≥ 1.5 and *p* ≤ 0.05), and the red dots represent the upregulated miRNA expression (fold change ≥ 1.5 and *p* ≤ 0.05). The grey dots have no statistical significance (*p* > 0.05). The green dots show a not obvious difference (fold change < 1.5 and *p* < 0.05).

A heat map was used to display all the differentially expressed miRNAs between group V vs. C, group D vs. C, and group D + V vs. C (Figure 7a). The miRNA microarray results indicated that 21 miRNAs were differentially expressed between the control group and the virus infection groups; of these, there were 13 upregulated miRNAs and 8 downregulated miRNAs (Figure 7b,e). The comparison between group C and group D showed that there were 83 differentially expressed miRNAs, of which 27 were upregulated miRNAs and 56 were downregulated miRNAs (Figure 7c,e). There were 88 differentially expressed miRNAs (30 up-regulated miRNAs and 58 down-regulated miRNAs) between group C and group D + V (Figure 7d,e). The intersection of differentially expressed miRNAs between group V vs. C, group D vs. C, and group D + V vs. C was illustrated using a Venn chart (Figure 7f). The results showed that there were two differentially expressed miRNAs at the intersection of groups V, D, and D + V compared with group C, namely miR-146b and miR-29c. In addition, there were five differentially expressed miRNAs at the intersection of the group V vs. C and group D + V vs. C, which were found to be miR-146b, miR-29c, miR-465c, miR-187, and miR-327. 

**Figure 8:**
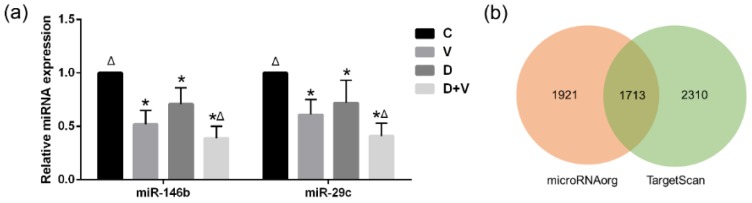
Differential miRNA validation and target prediction. (**a**) MicroRNA expression levels measured by RT-PCR. Data were corrected by the expression level of 18s RNA. The relative expression level was calculated by setting the control group expression level as 1. * *p* < 0.05 compared with group C; ^Δ^ *p* < 0.05 compared with group V. (**b**) Venn chart of the predicted target genes discovered by the TargetScan and microRNA.org databases.

PCR was used to validate the expression levels of miR-146b and miR-29c. Compared to group C, miR-146b exhibited a statistically significant decreasing trend in groups D and V, and the decreasing trend of miR-146b was even more significant in group D + V. Furthermore, miR-29c showed a decreasing trend after viral infection, which was significantly different from group C. Dysbacteriosis also caused a decrease in miR-29c expression, while it showed a more evident reduction in group D + V (Figure 8a). The trends of miRNAs expression measured by PCR were in accordance with the results of the miRNA microarray analysis. Furthermore, 1713 common target genes were discovered based on the intersections of the two databases (Figure 8b).

## 4. Discussion

In recent years, the relationship between the homeostatic maintenance of human health and the intestinal microbiota attracted increasing attention [14]. Perhaps the symbiotic relationship we share with the intestinal flora has indeed coevolved, as the coexistence of the host with intestinal flora represents an active and mutually beneficial relationship [15]. The healthy intestinal flora is suggested to be composed of a well-balanced community of three highly evolved groups of bacteria, which were termed symbionts, commensals, and pathobionts [9]. However, dysbiosis of intestinal flora may result in the loss of host–microbiota symbiosis, which often leads to a shift from symbiont to pathobiont. It means that an entire microbial community under the influence of specific conditions can tip the balance from homeostasis to destructive inflammation [16]. 

In this study, neomycin was used to establish the model of intestinal flora imbalance. Although the absorption of neomycin through the intact gastrointestinal mucosa is poor, it can significantly change the intestinal flora [17]. After neomycin treatment, abundance and species diversity of intestinal flora declined significantly. However, when virus infection was combined with dysbacteriosis, species diversity did not show a further decline, and even raised a little. When the species richness is the same, the greater the species evenness, and the greater the community diversity will be. As the species richness of intestinal flora shows a downward trend, the abnormality indicated that group D + V had a greater species evenness that group D. The higher species evenness indicated that the number of individual species in the community was relatively close. Combined with the species taxonomic results, it might be related to an outgrowth of potential pathogenic bacteria (pathobionts) and a decrease in the number of beneficial bacteria.

As a characteristic change in flora, the bacterial count of Bacteroidaceae was elevated in both group V and group D, and the greatest elevation was shown in group D + V. Bacteroidaceae contains some commensal bacteria with pathogenic potential, such as *Bacteroides fragilis*. *B. Fragilis* was found be able to promote regulatory T cell (Treg) function, and the phenomenon was associated with the capacity of this bacterium to limit T helper cell 17 (Th17) responses, which could suppress the immune response [18]. In addition, recent results suggested commensal microbes could induce constitutive and physiological inflammation through pattern-recognition receptors (PRRs) [19]. Nod2 is a receptor of nucleotide-binding oligomerization domain-like receptors (NLRs), which regulate the receptor-interacting protein 2 (RIP2)-dependent nuclear factor kappa B (NF-κB) pathway, and its deficiency was reported to lead to dysbiosis characterized by the outgrowth of Bacteroidetes [20]. Thus, the increase of Bacteroidaceae might be related to the weakened antiviral ability the under alteration of intestinal flora. Furthermore, antibiotic interference and virus infection significantly decreased the relative abundance of Desulfovibrionaceae, Helicobacteraceae, and Lachnospiraceae in the murine colon microbiota. Although neomycin treatment decreased the opportunistic pathogens (Desulfovibrionaceae), it destroyed the normal colony structure and species abundance. Therefore, the alteration of intestinal flora induced by antibiotics and the influenza virus not only reduced species abundance, but also destroyed the normal symbiotic relationship of the intestinal flora, including the reduction of beneficial bacteria (Lachnospiraceae) and an increase of opportunistic pathogen (Bacteroidaceae). 

Soner Yildiz found that influenza A virus (IAV) infection resulted in a reduction of commensal community richness, and also implicated that probiotics treatment could potentially balance the IAV-induced dysbiosis [21]. The changes in intestinal flora could be related to the abnormity of immune function and imbalance of immunologic homeostasis. In this study, higher viral load and lung index in the D + V group compared with group V were found. This phenomenon suggested that pulmonary virus infection could affect the intestinal microbiome; in turn, the imbalance of the intestinal flora could affect antiviral immunity in the lung and increase its susceptibility to influenza. 

MicroRNAs are a class of highly conserved noncoding single-stranded RNA molecules approximately 18–25 nucleotides in length, which play roles in the regulation of gene and protein expression at the post-transcriptional level [22]. As miRNAs participate in the modulation of both the innate and adaptive immune response, their abnormal expression may lead to disorder of the immune system [23]. Studies showed that miRNA plays important roles in the regulation of T-cell differentiation, the TLR7 signaling pathway, and the expression of inflammatory cytokines [24]. Thus, the decrease in host antivirus immune function might be related to the change in miRNAs. Based on the complex and precise physiological response of the human body, the relationship between intestinal flora and miRNA may be bidirectional. Williams et al. [25] raised three possible interactions between the host and gut microbiome involving miRNAs, which include miRNA regulation of host gene expression, influence of host miRNA expression by the gut microbiota, and influence of the gut microbiota through the release of miRNAs by the host. We sought to understand the role miRNA played in the interactions between intestinal flora and influenza infections.

Generally, it is believed that local microflora is more closely related to vicinal miRNA. However, the similar composition and structure of pulmonary flora based on 16S rRNA gene sequencing proved that neomycin had little effect on pulmonary flora. Since the oral administration of neomycin had almost no effect on the lung, it proved that it was not the local pulmonary bacterial flora changes that affected the expression of miRNAs. The miRNA microarray results showed that pulmonary miRNA expression was significantly changed after alteration of the intestinal flora. Therefore, it was the maladjusted intestinal flora that impacted the expression of pulmonary miRNAs. Combined with the important role of small RNAs in immunity, we speculate that the disorder of intestinal flora could affect the pulmonary immune response through miRNAs. After comparison and screening the differentially expressed miRNAs among the four groups, two common miRNAs were found: miR-146b and miR-29c. They are both related to immune response and might be suitable as therapeutic targets.

The miR-146 family includes miR-146a and miR-146b. Analysis of miR-146 gene expression unveiled its important role in response to a variety of microbial components and proinflammatory cytokines. Specifically, miR-146a was found to be an NF-κB-dependent gene, which could downregulate the protein levels of interleukin-1 receptor-associated kinase 1 (IRAK1) and tumor necrosis factor receptor-associated factor 6 (TRAF6) in Toll-like receptor signaling [26]. Moreover, Park et al. reported that miR-146a and miR-146b modulated DC apoptosis and cytokine production, which was inversely correlated with TRAF6 and IRAK1 expression [27]. In addition, our previous work revealed the negative effect of dysbacteriosis on the activation of the TLR7 signaling pathway and the release of type I interferon [11]. In this study, we found that miR-146a expression was reduced during viral infection, and it showed a more significant decreasing trend when viral infection was combined with dysbacteriosis. Therefore, under dysbacteriosis in influenza virus infection, miR-146a might work in connection with the suppression of TLR signaling pathway activation, which downregulates the host immune recognition mechanism-induced antiviral function.

The miR-29 family could affect helper T-cell differentiation and regulate type I interferon secretion to exert an antivirus role [28]. Ma et al. found that miR-29 expression was substantially downregulated in activated interferon gamma (IFN-γ)-producing natural killer (NK) cells and T-cell subsets [29]. Through the regulation of IFN-γ mRNA, miR-29 participated in the regulation of NK cell function and the Th1 cell response mechanism. As miR-29a and miR-29b were downregulated in IFN-γ secretion-type T cells, the increases in IFN-γ and Th1 inhibited the expression of miR-29. This study showed that, after viral infection, the expression level of miR-29c exhibited a decreasing trend. Combined with the previous studies, the mechanism might be associated with an increase in IFN-γ and the activation of NF-κB. Dysbacteriosis also led to a decrease in miR-29c. As observed in previous studies, the NF-κB expression in the dysbacteriosis group was higher than normal [11], and the associated inflammatory response might be responsible for the decrease in miR-29c. However, this trend was not significant. In addition, dysbacteriosis with viral infection also decreased the expression level of miR-29c. Thus, dysbacteriosis inhibited the expression of miR-29c during influenza virus infection, which could induce the enhancement of IFN-γ and NF-κB and result in a more serious inflammatory reaction.

Here, we only verified and analyzed miR-146b and miR-29c, which were found to have differential expression between group V vs. C, group D vs. C, and group D + V vs. C. Based on the discussion of their function, we believe that miR-146b and miR-29c might be good resources of resistance to influenza with intestinal flora alteration. However, the results of miRNA microarray analysis provided more information about the miRNAs and their target genes, which could help us find new approaches to fight influenza. For instance, miR-465c, miR-187, and miR-327 are also worth exploring under the effects of influenza and antibiotic abuse. As shown previously, miR-187 is a cancer-related microRNA, which was reported to play promoting or suppressive roles in different malignancies [30,31]. On the other hand, miR-327 was reported to indirectly suppress the TLR4 and TLR2 signaling pathways, and subsequently resulted in reduced myocardial infarct size and alleviated inflammation [32]. The results suggest that miR-465c, miR-187, and miR-327 could also serve as a promising therapeutic targets for influenza with or without intestinal flora alteration.

## 5. Conclusions

In conclusion, the alteration of intestinal flora affected the expression of pulmonary miRNAs and resulted in an enhancement of pulmonary influenza virus amplification. Owing to the important roles of miRNAs in the modulation of the host immune response, alteration of the intestinal flora induced by antibiotic interference might impede the activation of the innate immune defense system and interfere with host antiviral immunity through pulmonary miRNAs in influenza. Therefore, alteration of the intestinal flora stimulated pulmonary microRNAs to interfere with host antiviral immunity and reduced the host’s ability to kill the virus. These miRNAs, such as miR-146b and miR-29c, might be good resources of resistance to influenza under antibiotic abuse, and require further studies.
